# Plasma Exosomal miRNA Levels after Radiotherapy Are Associated with Early Progression and Metastasis of Cervical Cancer: A Pilot Study

**DOI:** 10.3390/jcm10102110

**Published:** 2021-05-13

**Authors:** Oyeon Cho, Do-Wan Kim, Jae-Youn Cheong

**Affiliations:** 1 Department of Radiation Oncology, Ajou University School of Medicine, Suwon 16499, Korea; 2 Ajou Translational Omics Center, Ajou University School of Medicine, Suwon 16499, Korea; dwkim@ajou.ac.kr (D.-W.K.); jaeyoun620@gmail.com (J.-Y.C.); 3 Department of Gastroenterology, Ajou University School of Medicine, Suwon 16499, Korea; 4 Human Genome Research & Bio-Resource Center, Ajou University Medical Center, Suwon 16499, Korea

**Keywords:** exosome, transcriptomics, fold change, cervical cancer, early progression, metastasis

## Abstract

Plasma exosomal miRNAs are key regulators of cell-cell interactions associated with several biological functions in patients with cancer. This pilot study aimed to investigate the log_2_ fold change (log_2_FC) of the expression of exosomal miRNAs and related mRNAs in the blood of patients with cervical cancer to identify prognostic markers better than those currently available. We sequenced plasma exosomal RNA from 56 blood samples collected from 28 patients with cervical cancer, who had been treated with concurrent chemoradiotherapy (CCRT). Changes in the expression of miRNAs and mRNAs before and after CCRT were represented as log_2_FC. Their biological functions were studied by miRNA-mRNA network analysis, using ingenuity pathway analysis, after the selection of two groups of miRNAs, each associated with early progression (EP) and metastasis, also described as initial stage. Seven patients experienced EP, three of whom died within four months after progression. Reduced levels of miR-1228-5p, miR-33a-5p, miR-3200-3p, and miR-6815-5p and increased levels of miR-146a-3p in patients with EP revealed unresolved inflammation, with accompanying increased expression of *PCK1* and decreased expression of *FCGR1A.* Increased levels of miR-605-5p, miR-6791-5p, miR-6780a-5p, and miR-6826-5p and decreased levels of miR-16-1-3p (or 15a-3p) were associated with the degree of metastasis and led to the systemic activation of myeloid, endothelial, and epithelial cells, as well as neurons, phagocytes, and platelets. Log_2_FCs in the expression of miRNAs and mRNAs from plasma exosomes after CCRT are associated with EP and metastasis, reflecting unresolved inflammation and systemic microenvironmental factors, respectively. However, this study, supported by preliminary data insufficient to reach clear conclusions, should be verified in larger prospective cohorts.

## 1. Introduction

The prognosis of cervical cancer is estimated based on the initial clinical stage; however, occasionally, patients with cervical cancer proceed to death earlier than expected. This may occur after three major cancer treatments: surgery, chemotherapy, and radiotherapy (RT) [1,2,3]. Such treatments destroy not only the cancer cells but also normal tissues and cause inflammation thereafter. Cancer development is deeply related to chronic inflammation [4], and patients undergoing treatments for cancer are subjected to higher psychological stress than usual [5]. Therefore, patients with cancer are also exposed to stress-derived inflammation. Notably, inflammation following tissue damage is essential for the maintenance of local and systemic homeostasis, autonomic nervous system, and the hypothalamic-pituitary-adrenal (HPA) axis [6]. Non-homeostatic states are associated with the co-existence of pro-inflammatory and anti-inflammatory signals, which may result in the abundant secretion of growth factors, high blood glucose levels, and inactivated cell-mediated immunity. In fact, such a systemic environment can be favorable for cancer progression; however, the association between unresolved inflammatory responses and cancer progression or death still remains undefined.

A 27% difference in 5-year progression-free survival has been reported between patients with cervical cancer and pelvic lymph node (LN) metastasis (2018 International Federation of Gynecology and Obstetrics (FIGO) stage IIIC1) and those with cervical cancer and para-aortic LN (PALN) metastasis (2018 FIGO IIIC2) [7]. Therefore, PALN metastasis is important for predicting clinical outcomes and establishing treatment strategies. Biologically, bone marrow-derived cells, lymphatic endothelial cells, and neuron cells are recruited to tumors to promote invasion and metastasis [8]. The same microenvironment may be formed at metastatic sites. Therefore, it may be useful to quantify systemic tumor microenvironment indicators for prognostic estimation.

The prediction of clinical outcomes in patients with cancer via analysis of the expression of individual genes has several limitations. First, selection of genes with a significantly different expression in group comparisons (e.g., healthy controls vs. patients with cancer and non-recurrence vs. recurrence) is usually performed. However, this analysis excludes pivotal regulators of various biological functions with small expression changes; moreover, it may be difficult to find all pivotal regulators even in a large population, because a plurality of upstream regulatory genes is likely to affect clinical outcomes. Second, gene expression levels may be affected by different treatment modalities. Therefore, comparing treatment outcomes may lead to the “dilution” of important variables, particularly when treatment groups are heterogeneous. Third, association studies of gene expression and clinical outcomes usually focus on the tumor and its microenvironment. However, clinical outcomes may also depend on different systemic responses. Therefore, access to blood is necessary to comprehensively investigate the systemic responses (of both cancer and normal cells) to treatment. Fourth, it is essential to analyze the highest number of regulatory genes related to cell-to-cell signaling for an efficient integration between numerous complex biological data and clinical results.

miRNA levels in plasma exosomes should be considered in this regard. The 30–100 nm extracellular vesicles, released by both cancer and normal cells, may regulate systemic biological functions relevant to clinical outcomes [9]. Therefore, in this study, we aimed to perform transcriptomic analysis of plasma exosomes isolated from the blood of patients with cervical cancer before treatment and in the second week after cisplatin-based concurrent chemoradiotherapy (CCRT). We calculated the log_2_ fold change (log_2_FC) values between the two samples from each patient to identify miRNAs as predictors of early progression (EP) and metastasis. Due to the use of network analysis tools, our findings provide mechanistic insights into EP and metastasis as well.

## 2. Methods

### 2.1. Patients

Two sets of 5–10 mL blood samples from 29 patients diagnosed with FIGO IB-IVB cervical cancer and treated with CCRT at the Department of Radiation Oncology, Ajou University Hospital from June 2018 to November 2019 were stored at the Biobank of Ajou University Hospital, a member of Korea Biobank Network, after the acquisition of informed consent from the patients (institutional review board approval number: BMR-SMP-18-248). The 58 samples were obtained before treatment and after the second week of CCRT, based on previous retrospective studies [10,11]. Plasma exosomal RNA sequencing and profiling were conducted by Macrogen (www.macrogen.com, Supplementary Methods). One patient was excluded owing to the significantly low expression levels. Diagnosis was histologically confirmed by biopsy, and regional LN and distant metastases were evaluated by magnetic resonance imaging (MRI) and positron emission tomography-computed tomography (PET-CT). External beam radiotherapy (EBRT) was delivered using 10 MV photons to the pelvis or PALNs. The pelvic RT dose was 45 Gy, delivered in 25 fractions, with a simultaneous integrated boost of 120–130% to regional LN metastases. RT response was evaluated by MRI in the fourth week of pelvic EBRT (36–45 Gy in 20–25 fractions). A weekly cisplatin regimen (30–70 mg/m^2^) was administered for six cycles to all patients. Three patients with one or two distant metastatic lesions were treated with the following EBRT regime. The patient with a single lesion in the left lung was treated with stereotactic body radiation therapy using 48 Gy in four fractions, starting in the fourth week of CCRT. The patient with a left supraclavicular lesion was treated with conformal RT of 55 Gy in 22 fractions, starting in the first week of CCRT. The patient with both left supraclavicular and right axillary lesions was treated with conformal RT of 40 Gy in 10 fractions, starting in the third week of CCRT. Twenty-six patients underwent intracavitary brachytherapy (ICBT), except the one whose ICBT was replaced with EBRT boost and another who refused further treatment (Iridium-192; GammaMedplus iX, Varian, Palo Alto, CA, USA). Weekly cisplatin (30–70 mg/m^2^) was administered in 6 cycles during RT to all patients, and the latter were followed up every 1–3 months after treatment completion. Primary cervical tumors and regional LN and distant metastases were evaluated by pelvic examination, Pap smear test, tumor marker analysis, MRI, and CT.

### 2.2. Log_2_FC and Power Transformation for miRNA and mRNA

Next generation sequencing data from plasma exosomes included data of small RNAs, long non-coding RNAs, and mRNAs, from which miRNAs and mRNAs were used for analysis. After removal of the RNAs undetected in 50% of samples, 586 miRNAs and 15,324 mRNAs were analyzed. Log_2_FC values between read counts of miRNAs and mRNAs, before treatment (control) and after the second week of CCRT (treatment), were calculated after TMM normalization using edgeR. Reads per million (RPM) values before treatment were transformed to normal distribution using Box-Cox function to find the miRNAs most relevant to pretreatment. miR-16-1-3p or miRNAs with pretreatment RPM values were negatively correlated with log_2_FC values (Figure S1A).

### 2.3. Selection of RNAs to Predict Clinical End Points

The matrix of Pearson’s correlations between all RNAs was calculated by recorr function in Hmisc package for R programming. RNAs associated with EP (|R| > 0.4) and stage (|R| > 0, staging order and |R| > 0, extrapelvic metastasis) were primarily selected. The optimal model using selected RNAs was suggested by an exhaustive search of regsubsets in leaps package for R (Figures S2A and S5A). The sum and difference of RNAs in the suggested model were relevant to EP and stage by Wilcoxon rank-sum test and Kruskal–Wallis test, respectively (Figures S2B and S5B).

### 2.4. Network Analysis

Network analyses were performed using Prim’s algorithm of minimum spanning tree in igraph package for R (Figure S1). Positively and negatively correlated edges are shown in red and blue, respectively, and were calculated as Pearson’s correlations.

### 2.5. Ingenuity Pathway Analysis

The most significant diseases and bio-functions analyzed using ingenuity pathway analysis (IPA) software (QIAGEN, https://www.qiagenbioinformatics.com/products/ingenuity-pathway-analysis) were selected based on the activated Z-scores of downstream effects of the analysis [12]. Positive and negative Z-scores indicated promoted and inhibited functional activities, respectively.

Data analysis and visualization were performed using R version 3.6.3 (https://www.r-project.org).

## 3. Results

### 3.1. Early Progression and Tumor Stage

**Figure 1:**
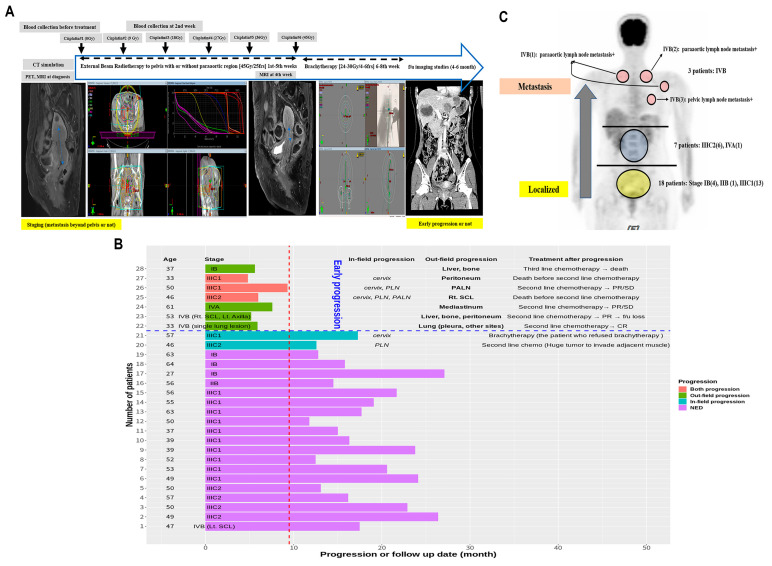
Transcriptomic analysis of miRNAs within exosomes isolated from the plasma of 28 patients with cervical cancer. (**A**) Clinical endpoints and blood sampling timeline. (**B**) Bar graph of follow-up duration and description of early progression and second-line treatment. (**C**) Metastatic sites according to the 2018 International Federation of Gynecology and Obstetrics (FIGO) staging. One patient with bladder invasion (stage IVA) also showed para-aortic lymph node metastasis.

Two sets of blood samples were collected from 28 patients with cervical cancer before and two weeks after the initiation of CCRT. Treatment and follow-up data of EP and metastasis were collected (Figure 1A). EP was defined as the observation of new tumor(s) outside the RT field within a year after diagnosis; tumor progression within the RT field or after one year of diagnosis was not defined as EP. The treatment approaches and cancer progression results are shown in Figure 1B. Considering a median follow-up of 16.9 months, seven patients showed EP; three of them died within four months after progression, three showed responses to second-line chemotherapy and are still being followed-up, and the remaining patient discontinued follow-up. Additionally, two patients showed loco-regional progression; one of them had cervical lesion progression after refusal of ICBT, whereas the other showed left pelvic LN lesion progression despite irradiation (more than 70 Gy). Clinical characteristics of the patients are described in Table 1. According to FIGO staging, the lesions of 18 patients with stage IB-IIIC1 were localized in the pelvis, whereas those of patients with stage IIIC2-IVA spread to the PALNs and those of patients with stage IVB spread to the lungs and supraclavicular and axillary LNs (Figure 1C). This clearly showed that tumor staging reflects the degree of metastasis.

### 3.2. Selection of miRNAs That Predicted Early Progression and Tumor Stage Better

**Figure 2:**
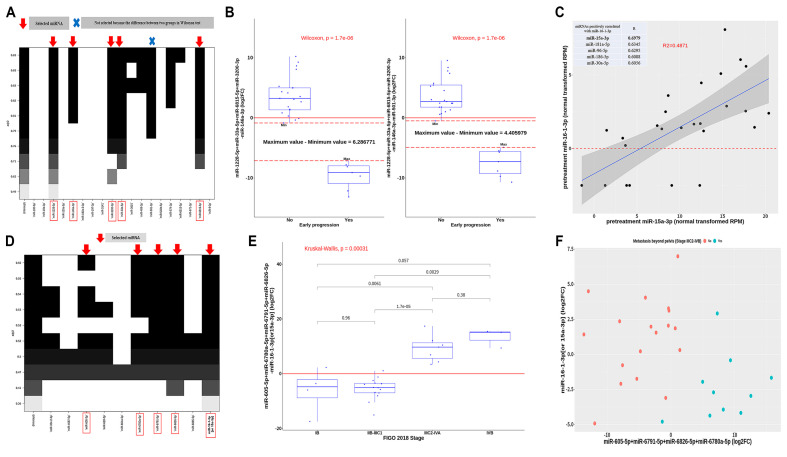
Selection of miRNAs associated with early progression and stage in cervical cancer. (**A**) Five miRNAs, selected after obtaining adjusted R values from multiple regressions of all possible combinations of 19 miRNAs, were significantly associated with early progression. (**B**) In two groups related to early progression status, the difference of sum and subtraction of the selected 5 miRNAs was greater than that of the six selected miRNAs. (**C**) Pre-treatment normal transformed miR-16-1-3p correlated better with miR-15a-3p among all miRNAs. (**D**) Five miRNAs, selected after obtaining adjusted R values from multiple linear regressions of all possible combinations of 10 miRNAs, were significantly associated with stage. (**E**) Sum and difference of the selected 5 miRNAs were positively correlated with stage. (**F**) Correlation between miR-605-5p+miR-6791-5p+miR-6826-5p+miR-6780a-5p, miR-16-1-3p (or 15a-3p), and extrapelvic metastasis.

miR-1228-5p, miR-146a-3p, miR-33a-5p, miR-3200-3p, miR-501-3p, and miR-6815-5p were found to be associated with EP (Figure 2A). We also performed a multiple linear regression (MLR) analysis of the selected miRNAs; the difference in expression of these miRNAs between the two groups, according to EP, was larger for five miRNAs (excluding miR-501-3p) than all the above six miRNAs (Figure 2B). Therefore, miR-1228-5p, miR-146a-3p, miR-33a-5p, miR-3200-3p, and miR-6815-5p were selected as predictors of EP.

Only miR-16-1-3p, known as a cluster of miR-15a [13], was negatively correlated with tumor stage (R = −0.488, and −0.546 for staging order and extrapelvic metastasis, respectively). Considering pretreatment RPM values, miR-16-1-3p was most positively correlated with miR-15a-3p (Figure 2C). Since miR-16-1-3p was not detected in four patients, it was replaced with miR-15a-3p to obtain miR-16-1-3p (or 15a-3p) (log_2_FC). Importantly, miR-605-5p, miR-6791-5p, miR-6780a-5p, miR-6826-5p, and miR-16-1-3p (or 15a-3p) were associated with stages (Figure 2D). After MLR analysis, stage was found to be significantly correlated with miR-605-5p+miR-6791-5p+miR-6780a-5p+miR-6826-5p-miR-16-1-3p (or 15a-3p). Additionally, extrapelvic metastasis was negatively correlated with miR-16-1-3p (or 15a-3p) while being positively correlated with miR-605-5p+miR-6791-5p+miR-6780a-5p+miR-6826-5p (Figure 2F).

### 3.3. Selection of RNAs According to Disease and Biological Functions Using IPA

**Figure 3:**
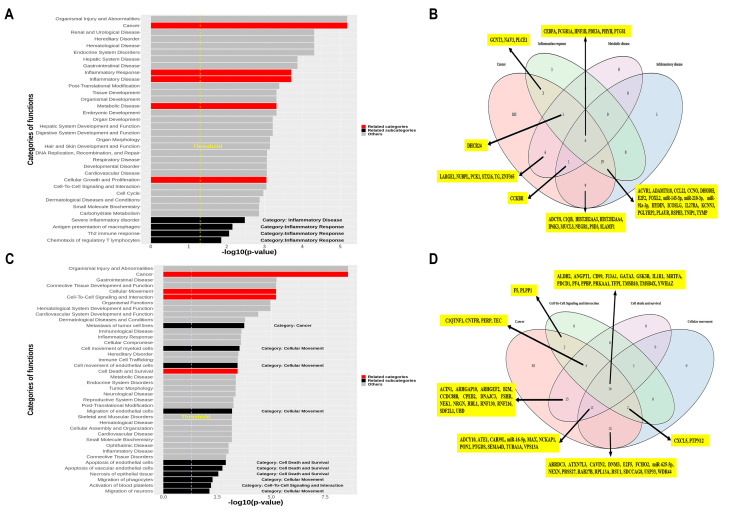
Ingenuity pathway analysis-based selection of RNAs using functional categories related to early progression and stage. (**A**) (Sub)categories for early progression are sorted by relevance. Five categories and four subcategories are selected based on the assumption of uncontrolled inflammation. (**B**) Venn diagram representing four categories; RNAs overlapping with the cancer category are highlighted. (**C**) (Sub)categories for stage are sorted by relevance. Four categories and 10 subcategories are selected based on the assumption of the correlation between tumor microenvironment and metastasis. (**D**) Venn diagram representing four categories; RNAs overlapping with the cancer category are highlighted.

To understand the function of miRNAs associated with EP and stage, IPA was performed after identification of the network structure formed by the selected miRNAs and adjacent RNAs (Figure S1). Table S1 shows the candidate RNAs, including mRNAs, intimately associated with three clinical endpoints and involved in miRNA-mRNA networks related to EP and stage (Figure S1B,D). Importantly, cancer, inflammatory response (IR), inflammatory disease (ID), metabolic disease (MD), and cellular growth and proliferation (CGP) were selected, among the top 30 categories, to be associated with EP; subcategories relevant to ID or IR were also defined in terms of EP (Figure 3A,B). Additionally, cancer, cellular movement (CM), cell-to-cell signaling and interaction (CCSI), and cell death and survival (CDS) were selected, among the top 30 categories, to be associated with stage (Figure 3C,D).

### 3.4. Association between Unresolved Inflammation and Early Progression in miRNA-mRNA Simplified Network Analysis

**Figure 4:**
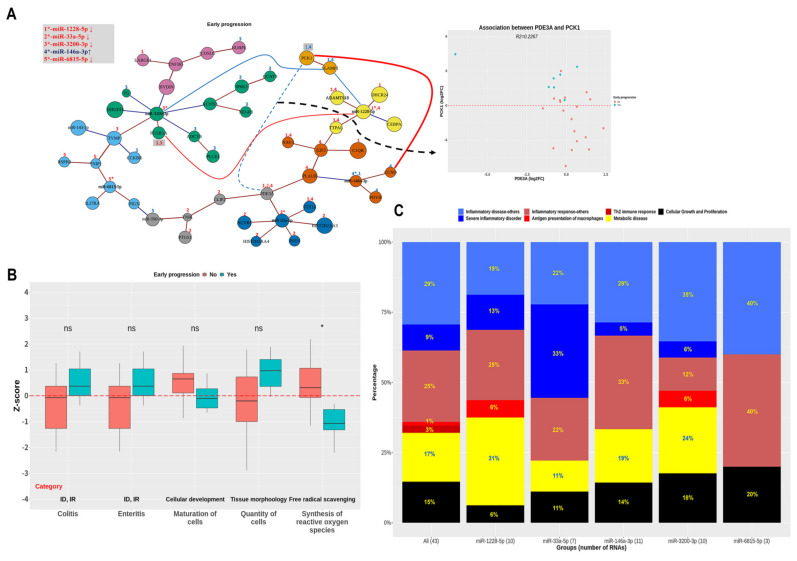
Simplified network of miRNA-mRNA interactions in early progression. (**A**) A simplified network is presented from 10 RNAs involved in the shortest pathway connecting 5 mRNAs and 45 RNAs within four functional categories related to early progression. RNAs altered by the five main miRNAs are displayed by number(s) above vertices in red (downregulation) or blue (upregulation), and in bold (*p* < 0.05) or plain (*p* ≥ 0.05) fonts. Red and blue edges correspond to positive and negative correlations, respectively. (**B**) Subcategories are displayed to show the difference in Z-scores according to early progression; subcategories were selected based on significant Z-scores in all patients using the log_2_FC values of all 48 RNAs included in this network. Functional categories are defined below the boxplots as ID (inflammatory disease), and IR (inflammatory response). ns, *p* ≥ 0.05; *, *p* < 0.05. (**C**) Diseases and biological functions associated with the 43 RNAs and five groups formed by primary miRNAs in the network are shown using boxplots. Statistical analysis was performed using the Wilcoxon rank-sum test or Kruskal–Wallis test. Downregulation and upregulation of RNAs refer to log_2_FC < −1.5 and log_2_FC > 1.5, respectively.

The simplified network represented in Figure 4A was constructed with the RNAs, including the shortest distance-connecting five main miRNAs represented in Figure S1B, as well as the RNAs selected using IPA (Figure 3B). The number of RNAs altered by miR-1228-5p, miR-146a-3p, and miR-3200-3p was 10–11, whereas that altered by miR-33a-5p and miR-6815-5p was 7 and 2, respectively; additionally, there were nine overlapping cases among the RNAs changed by miR-1228-5p and miR-146a-3p. The network suggested that *PCK1* is upregulated by *CCNO* following the upregulation of miR-146a-3p, or by *SLAMF1* following the downregulation of miR-1228-5p. In addition, the downregulation of miR-1228-5p and upregulation of miR-146a-3p were interrelated. Moreover, the changes in miR-1228-5p, miR-33a-5p, and miR-146a-3p according to EP were associated with the upregulation of *PCK1* following the downregulation of *PDE3A*. However, the downregulation of miR-3200-3p was not correlated with the upregulation of *PCK1*, despite being relevant to the upregulation of *SLAMF1* (R^2^ = 0.3805). Additionally, the downregulation of miR-1228-5p and miR-3200-3p was associated with the downregulation of *FCGR1A*; correlation was higher for miR-3200-3p (R^2^ = 0.4939) than for miR-1228-5p (R^2^ = 0.2227). Curiously, *PCK1* and *FCGR1A*, the best predictors of EP among all mRNAs in Figure 4A (Figure S2), replaced miR-146a-3p and miR-3200-3p, respectively, when they were combined with the main miRNAs towards high adjusted R (Figure S2B). This supported the idea that primary miRNAs are involved in EP through *PCK1* and *FCGR1A*. The boxplots of RNAs altered by main miRNAs are presented in Figure S3A; the changes in RNA expression due to miR-33a-5p or miR-6815-5p were mostly between −1.5 and 1.5 log_2_FC, whereas that due to miR-1228-5p, miR-146a-3p, or miR-3200-3p were not. Therefore, miR-1228-5p, miR-146a-3p, and miR-3200-3p were more relevant to EP than miR-33a-5p and miR-6815-5p, considering the association between these miRNAs and *PCK1* or *FCGR1A*, the number of RNAs they regulate, and their relevant changes.

The difference in Z-scores according to EP can be interpreted as increased pro-inflammation (colitis↑, enteritis↑), anti-inflammation (synthesis of ROS↓), and CGP (maturation of cells↓, quantity of cells↑) in the EP group (Figure 4B). All RNAs were related to ID (38%), IR (29%), MD (17%), and CGP (15%) (Figure 4C). Moreover, three groups of RNAs regulated by miR-1228-5p, miR-146a-3p, and miR-3200-3p were relatively relevant to MD and a group of RNAs regulated by miR-33a-5p was related to severe inflammatory disorders; additionally, two groups of RNAs regulated by miR-3200-3p and miR-6815-5p were relatively associated with CGP, whereas two groups regulated by miR-3200-3p and miR-1228-5p included *FCGR1A*, relevant to antigen presentation in macrophages. The functions of mRNAs altered by the five miRNAs are described as references (Table 2). Importantly, all miRNAs were relevant to increased pro-inflammation or cancer progression; four miRNAs (except miR-6815-5p) were associated with increased anti-inflammation, and miR-1228-5p, miR-146a-3p, and miR-3200-3p were linked to reduced cell-mediated immunity or increased blood glucose.

Cortisol is a representative anti-inflammatory hormone associated with the weakening of immune responses and gluconeogenesis. Considering the HPA axis in the central nervous system and peripheral tissues [14], an association between *CRH*, *POMC*, *CYP11B1*, *HSD11B1*, and the primary miRNAs is presented in Figure S3B. *CYP11B1* and *HSD11B1* are mRNAs relevant to the production of cortisol. Importantly, a network constructed by *CRH*, *POMC*, *CYP11B1*, *HSD11B1*, *CYP11B1*+*HSD11B1*, and miR-3200-3p revealed the latter to be a mediator between *CRH* and *CYP11B1*.

The simplified network may be divided into two groups: a group formed by miR-1228-5p, miR-146a-3p, and miR-33a-5p centralized by *PDE3A*, and another formed by miR-6815-5p and miR-3200-3p. Importantly, early death was associated with dysregulation of all miRNAs in both groups (|1.5| > log_2_FC) according to EP (Figure S3C).

### 3.5. Association between Systemic Tumor Microenvironment and Metastasis in miRNA-mRNA Simplified Network Analysis

**Figure 5:**
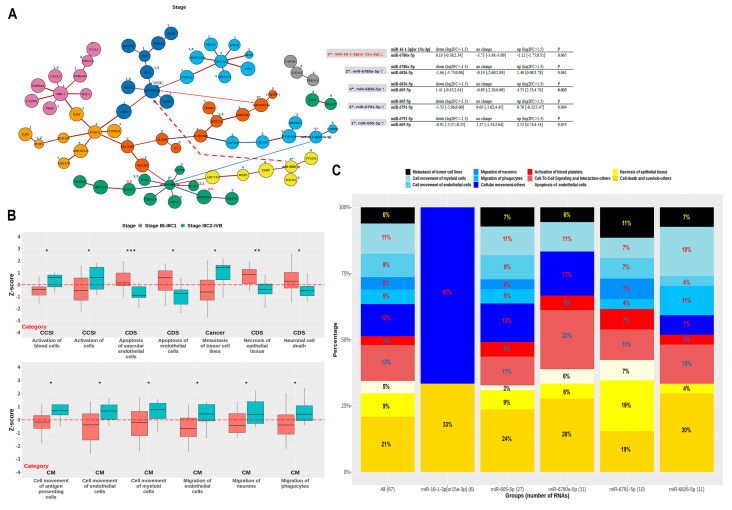
Simplified network of miRNA-mRNA interactions in metastasis. (**A**) A simplified network is presented from 15 RNAs involved in the shortest pathway connecting 5 mRNAs and 65 RNAs within four functional categories related to metastasis. RNAs changed by the five main miRNAs are displayed by number(s) above the vertices in red (downregulation) or blue (upregulation), and in bold (*p* < 0.05) or plain (*p* ≥ 0.05) fonts. Red and blue edges correspond to positive and negative correlations, respectively. (**B**) Subcategories show the difference in Z-scores according to extrapelvic metastasis; subcategories were selected based on significant Z-scores in all patients using the log_2_FC values of all 72 RNAs included in this network. * *p* < 0.05, ** *p* < 0.01, *** *p* < 0.001. (**C**) Biological functions associated with the 67 RNAs and five groups formed by primary miRNAs in the network are shown using boxplots. Statistical analysis was performed using the Wilcoxon rank-sum test or Kruskal–Wallis test. Downregulation and upregulation of RNAs refer to log_2_FC < −1.5 and log_2_FC > 1.5, respectively.

The simplified network represented in Figure 5A was constructed with the RNAs including the shortest distance-connecting five main miRNAs represented in Figure S1D and those selected using IPA (Figure 3D). The boxplots of RNAs altered by the five main miRNAs showed the extent to which the RNAs were altered by each primary miRNA, apart from the statistically significant changes, as well as which (sub)categories they belonged to (Figure S4). Downregulation, no change, and upregulation of miR-16-1-3p (or 15a-3p) were relevant to the upregulation, downregulation, and no change of miR-6780a-5p, respectively. This resulted from two outliers of negative linear correlation between miR-16-1-3p (or 15a-3p) and miR-6780a-5p. However, miR-6780a-5p was positively correlated with miR-6826-5p and positively relevant to miR-605-5p, which in turn was positively related to miR-6791-5p. While the number of RNAs altered by miR-605-5p was the highest (27), those altered by miR-6780a-5p, miR-6791-5p, and miR-6826-5p were similar (10–11); moreover, the number of RNAs changed by miR-16-1-3p (or 15a-3p) was the lowest at 6. Therefore, miR-16-1-3p (or 15a-3p) might regulate the other four miRNAs, whereas miR-605-5p might be regulated by three other miRNAs (except miR-6791-5p). *FAM168A*, *RBP3*, and *C1QTNF1* were the best mRNA predictors for stage (Figure S5). Additionally, *RBP3* and *C1QTNF1* replaced miR-6826-5p and miR-6791-5p, respectively, when they were combined with the five main miRNAs toward high adjusted R (Figure S5B). Therefore, *RBP3* and *C1QTNF1* may be the key mRNAs relevant to miR-6826-5p and miR-6791-5p, respectively.

The difference in Z-scores according to extrapelvic metastasis suggested that all of the systemic angiogenesis, tumor-associated macrophages, neurogenesis, and blood cell activation contribute to metastasis (Figure 5B); all RNAs were related to CM (42%), CCSI (16%), CDS (35%), and metastasis of tumor cell lines (6%) (Figure 5C). Importantly, four groups of RNAs altered by miRNAs (except miR-16-1-3p (or 15a-3p)) were relevant to CM of myeloid cells, activation of blood platelets, and necrosis of epithelial cells. In addition, both miR-605-5p and miR-6791-5p were associated with the CM of both neurons and phagocytes and with the apoptosis of endothelial cells; their functions were also similar. However, miR-6826-5p was not implicated in the migration of neurons and apoptosis of endothelial cells; it was involved in the CM of myeloid cells and phagocytes with the highest magnitude across all groups. In fact, miR-6780a-5p was the one with the lowest number of biological functions among all groups. These data together suggested that miR-16-1-3p (or 15a-3) has rare active functions, whereas the four groups of RNAs altered by other miRNAs have active functions relevant to myeloid, endothelial, and epithelial cells, as well as to neurons, phagocytes, and platelets.

## 4. Discussion

Our findings clearly suggested that the log_2_FC of miRNAs contained within plasma exosomes are associated with EP and metastasis in patients with cervical cancer. Importantly, the biological functions of mRNAs regulated by these miRNAs supported the association of unresolved inflammation with EP, systemic tumor microenvironment, and metastasis.

The balance between pro-inflammatory and anti-inflammatory states is essential for inflammatory homeostasis [6,49]. However, anti-inflammatory cytokines and hormones cannot efficiently reduce inflammation if the balance is disturbed. In fact, this is probably what happens in cancer; the persistence of pro-inflammatory cytokines results in cancer proliferation [50]. In this study, we showed that the changes in mRNAs altered by miR-1228-5p, miR-146a-3p, miR-33a-5p, miR-3200-3p, and miR-6815-5p in patients with EP are associated with loss of homeostasis and simultaneous induction of pro-inflammation, anti-inflammation, gluconeogenesis (*PCK1*), and immune suppression (*FCGR1A*) (Figure 4 and Figure S2). This unresolved inflammation is consistent for patients with EP, who require more dexamethasone for enteritis or cystitis after completion of RT (Table 1) than those without EP. Moreover, the downregulation of miR-3200-3p was relevant to the upregulation of both *CRH* and *CYP11B1* (Figure S3B) and supported the association between EP and dysregulation of the peripheral HPA axis. Importantly, for EP, we found two relevant miRNA groups, including one formed by miR-1228-5p, miR-146a-3p, and miR-33a-5p, and another by miR-3200-3p and miR-6815-5p. The failure of regulation of all miRNAs in each group was associated with early death after EP (Figure S3C). This implied that miR-1228-5p and miR-146a-3p are complementary to miR-33a-5p, whereas miR-3200-3p is complementary to miR-6815-5p. The novel findings clearly supported the notion that cancer progression during conventional treatments results from the dysregulation of five miRNAs related to inflammatory responses.

Tumors recruit various types of stromal cells, such as myeloid, endothelial, and epithelial cells, as well as neurons, macrophages, lymphocytes, and platelets, which induce metastasis and affect survival, progression, angiogenesis, and immune evasion [8]. Our results revealed that expression levels of four miRNAs associated with stromal cells are increased in metastasis, and are negatively correlated with miR-16-1-3p (or 15a-3p) (Figure 5). Additionally, two mRNAs, changed by miR-6826-5p and miR-6791-5p, were also important to the tumor stage. *RBP3*, associated with the CM of myeloid cells and phagocytes, and *C1QTNF1*, associated with the activation of platelets, played key roles towards metastasis in the tumor microenvironment (Figures S4 and S5). In fact, such factors might affect cell signaling and consequent activation of stromal cells following tumor metastasis outside the RT field. Therefore, quantification of these miRNAs could reflect the metastatic microenvironment not seen otherwise in MRI or CT scans, thereby helping the establishment of customized treatment strategies according to the risk. Furthermore, our results suggested that miR-16-1-3p (or 15a-3p) may be a potential upstream regulator important for the suppression of metastasis by modulation of the tumor microenvironment. Its role as a tumor suppressor in cancer supported the potential of miR-16-1-3p (or 15a-3p) as a therapeutic target in cervical cancer [13].

In this study, we used the sample obtained 2 weeks after CCRT to calculate log_2_FC for the following reasons. A previous study reported that lymphopenia during CCRT is relevant to cervical cancer prognosis [51]. Furthermore, it has been reported that hematological parameters, including lymphocyte count, at 2 weeks during pelvic CCRT could predict treatment outcome [10,11]. A sharp decline in lymphocyte count (poor radiation tolerance of lymphocytes) was more associated with clinical results than a gradual decline (good radiation tolerance of lymphocytes) during 2 weeks post CCRT in locally advanced cervical cancer [52]. This lymphopenia may result from DNA damage in lymphocytes by CCRT, an immune-response escape mechanism of cancer, and intrinsic factors, such as inappropriate inflammatory response or suppressed innate immunity against CCRT. We hypothesize that 2 weeks post CCRT is the most appropriate period to evaluate the association between intrinsic factors and EP of cervical cancer because the influence of CCRT on circulating lymphocytes and cancer may increase with treatment progression.

Han et al. compared miRNA expression profiles of advanced stage cervical squamous cell carcinoma between patients resistant (EP) and those sensitive to CCRT for screening candidate miRNAs [53]. In the present study, we evaluated the association between log_2_FC of miRNAs in each patient according to irradiation and EP. The previous study by Han et al. was a case-control study, whereas ours is a cohort study. A case-control study may have a selection bias despite careful individual matching, and it is difficult to identify factors that are relatively less relevant to EP, whereas a cohort study has the advantage to integrate various risk factors. Therefore, serum miRNA-206 screened in the previous study might not have been upregulated in all patients who showed EP, but largely upregulated in some patients who showed EP. In the present study, we considered log_2_FC of each miRNA as a risk factor in this small cohort, and several log_2_FCs of miRNAs were clearly divided into two groups according to EP without overlapping of boxplots as shown in Figure 2B. In addition, IPA using log_2_FCs of mRNAs correlated with these groups revealed a possible biological background. We used a research method different from that used in previous studies, in that it enables cohort studies. We used next generation sequencing of miRNA from plasma exosomes to maximize this advantage. We believe that this method can be used to screen potential miRNAs associated with clinical results more efficiently and accurately than previous methods.

## 5. Conclusions

The log_2_FCs of miRNAs and mRNAs from plasma exosomes were found to be associated with unresolved inflammation and microenvironmental factors that trigger metastasis. The estimated biological functions of the main miRNAs supported their associations with clinical outcomes. In addition, the study showed plasma exosomes to be useful tools for addressing interesting biological problems in oncology, and be potential clinical biomarkers for patients with cervical cancer.

Nevertheless, this study involved a small number of patients, which is a clear limitation in this preliminary step to lay a foundation to predict EP and metastasis in patients with cervical cancer treated with CCRT. Therefore, the methods and findings of this study should be verified in larger prospective cohorts.

## Figures and Tables

**Table 1:** Clinical characteristics of the patients.

	All	Early Progression		*p*
	(N = 28)	No (N = 21)	Yes (N = 7)	
Age (years) (IQR)	50.0 (42.5;56.0)	50.0 (47.0;56.0)	46.0 (35.0;51.5)	0.184
FIGO staging 2018, *n* (%)				0.298
- IB	4 (14.3%)	3 (14.3%)	1 (14.3%)	
- IIB-IIIC1	14 (50.0%)	12 (57.1%)	2 (28.6%)	
- IIIC2-IVA	7 (25.0%)	5 (23.8%)	2 (28.6%)	
- IVB	3 (10.7%)	1 (4.8%)	2 (28.6%)	
Pathology, *n* (%)				0.017
- Adenocarcinoma	4 (14.3%)	1 (4.8%)	3 (42.9%)	
- Adenosquamous cell carcinoma	1 (3.6%)	0 (0.0%)	1 (14.3%)	
- Unclassified carcinoma	1 (3.6%)	1 (4.8%)	0 (0.0%)	
- Squamous cell carcinoma	22 (78.6%)	19 (90.5%)	3 (42.9%)	
RT field, *n* (%)				0.815
Pelvis	19 (67.9%)	15 (71.4%)	4 (57.1%)	
Pelvis with para-aortic region	9 (32.1%)	6 (28.6%)	3 (42.9%)	
Total dose (EQD2) (IQR)	76.2 (72.2;84.2)	75.5 (72.2;84.2)	84.2 (74.2;84.2)	0.357
Intracavitary brachytherapy, *n* (%)				0.483
- No treatment	2 (7.1%)	1 (4.8%) Refusal	1 (14.3%) EBRT	
- 24 Gy in four fractions	10 (35.7%)	9 (42.9%)	1 (14.3%)	
- 24 Gy in six fractions	5 (17.9%)	4 (19.0%)	1 (14.3%)	
- 25 Gy in five fractions	1 (3.6%)	1 (4.8%)	0 (0.0%)	
- 30 Gy in six fractions	10 (35.7%)	6 (28.6%)	4 (57.1%)	
Dexamethasone during RT, *n* (%)				1.000
No	21 (75.0%)	16 (76.2%)	5 (71.4%)	
Yes	7 (25.0%)	5 (23.8%)	2 (28.6%)	
Dexamethasone after RT, *n* (%)				0.061
No	24 (85.7%)	20 (95.2%)	4 (57.1%)	
Yes	4 (14.3%)	1 (4.8%)	3 (42.9%)	
Death, *n* (%)				0.014
No	25 (89.3%)	21 (100.0%)	4 (57.1%)	
Yes	3 (10.7%)	0 (0.0%)	3 (42.9%)	

RT, radiotherapy; EQD2, equivalent dose in 2 Gy fractions; FIGO, International Federation of Gynecology and Obstetrics; IQR, interquartile range.

**Table 2:** Biological functions of RNAs that changed significantly according to the upregulation or downregulation of the five main miRNAs are reviewed focusing on the inflammatory response.

Regulatory miRNAs	mRNAs	Related Function	References
Pro-inflammation			
miR-1228-5p↓, miR-33a-5p↓, miR-146a-3p↑	PDE3A↓	Cardiac contractility↑ Vascular contractility↑	[15]
miR-1228-5p↓, miR-146a-3p↑	ADAMTS-18↓	Platelet activation↑	[16]
miR-3200-3p↓	TG↑	Inflammatory cytokine↑ Cancer proliferation↑	[17,18]
miR-33a-5p↓	HIST2H2AA3/4↓	DNA damage↑	[19]
miR-3200-3p↓	PLCE1↑	Inflammatory cytokine↑ Cancer proliferation↑	[20]
miR-3200-3p↓	GCNT3↑	Inflammatory cytokine↑	[21,22]
miR-146a-3p↑	PHYH↑	Peroxisome ↑	[23]
miR-6815-5p↓	TNIP1↓	Anti-inflammation↓	[24]
miR-6815-5p↓	RSPH3↓	Inflammatory cytokine↑	[25,26]
Anti-inflammation			
miR-1228-5p↓, miR-33a-5p↓, miR-146a-3p↑	PDE3A↓	Platelet aggregation↓	[15]
miR-146a-3p↑	PLAUR↓	Plasminogen activation↓	[27]
miR-33a-5p↓	PTGS1↓	Prostaglandins↓ -> anti-inflammation↑	[28]
miR-1228-5p↓	DHCR24↓	Inflammatory gene expression↓	[29]
miR-146a-3p↑	E2F2↓	Inflammatory signal↓	[30]
miR-3200-3p↓	CCKBR↑	Vagus nerve stimulation -> anti-inflammation↑	[31]
Cell mediated immunity↓			
miR-1228-5p↓, miR-146a-3p↑	SLAMF1↑	Activation of macrophages↓	[32]
miR-1228-5p↓, miR-3200-3p↓	FCGR1A↓	Antigen presentation↓	[33]
miR-1228-5p↓	C1QB↓	Antigen presentation↓	[34]
Blood glucose↑			
miR-3200-3p↓	NUBPL↑	Mitochondrial complex 1↑ -> Blood glucose↑	[35,36]
miR-1228-5p↓, miR-146a-3p↑	PCK1↑	Blood glucose↑	[37]
miR-1228-5p↓, miR-146a-3p↑	STX16↓	Intracellular glucose transport↓	[38]
miR-3200-3p↓	ADCY8↑	Obese and type 2 diabetes	[39]
miR-3200-3p↓	IP6K3↑	Blood glucose↑	[40]
miR-3200-3p↓	NEGR1↑	Obese and insulin resistance↑	[41]
Cancer progression			
miR-1228-5p↓, miR-146a-3p↑	NAV3↓	Cancer metastasis↑	[42]
miR-1228-5p↓	LARGE1↓	Cancer metastasis↑	[43]
miR-33a-5p↓	PSD3↓	Cancer proliferation↑	[44]
miR-146a-3p↑	CCNO↑	Cancer proliferation↑	[45]
miR-6815-5p↓	miR-590-3p↑	Cancer progression↑	[46]
Unclassified			
miR-33a-5p↓	ACVR1↓	Oncogene vs. tumor suppressor gene	[47]
miR-3200-3p↓	TYMP↓	Cancer proliferation↓ vs. chemo response↓	[48]

↑; Increase, ↓; Decrease.

## Data Availability

All data analyzed during this study are available in the following. Original sequencing data: ArrayExpress (accession number: E-MTAB-10215) Coding and dataset: https://data.mendeley.com/datasets/z9f4ydxs9m/draft?a=40df002d-7286-4be7-af4c-09c40bff094b.

## Supplementary Materials

The following are available online at https://www.mdpi.com/article/10.3390/jcm10102110/s1, Supplementary Methods: Process of plasma exosomal RNA sequencing and profiling, Figure S1: Identification of the network structure formed by selected miRNAs and adjacent RNAs (related to Figure 3); Figure S2: Selection of mRNAs associated with early progression in a simplified network (related to Figure 4); Figure S3: Details of simplified network of miRNA-mRNA interactions in early progression (related to Figure 4); Figure S4: Details of simplified network of miRNA-mRNA interactions in metastasis (related to Figure 5); Figure S5: Selection of mRNAs associated with metastasis in the simplified network (related Figure 5), Table S1: Candidate RNAs for the ingenuity pathway analysis (related to Figure 3).
