# Supplementary material for: Plasma Exosomal miRNA Levels after Radiotherapy Are Associated with Early Progression and Metastasis of Cervical Cancer: A Pilot Study
Source: J Clin Med. 2021 May 13;10(10):2110. doi: 10.3390/jcm10102110 (PMC8153571; doi:10.3390/jcm10102110)
Supplement: Supplementary file 1 [file jcm-10-02110-s001.zip › Supplementary_figures_and_tables.pdf]

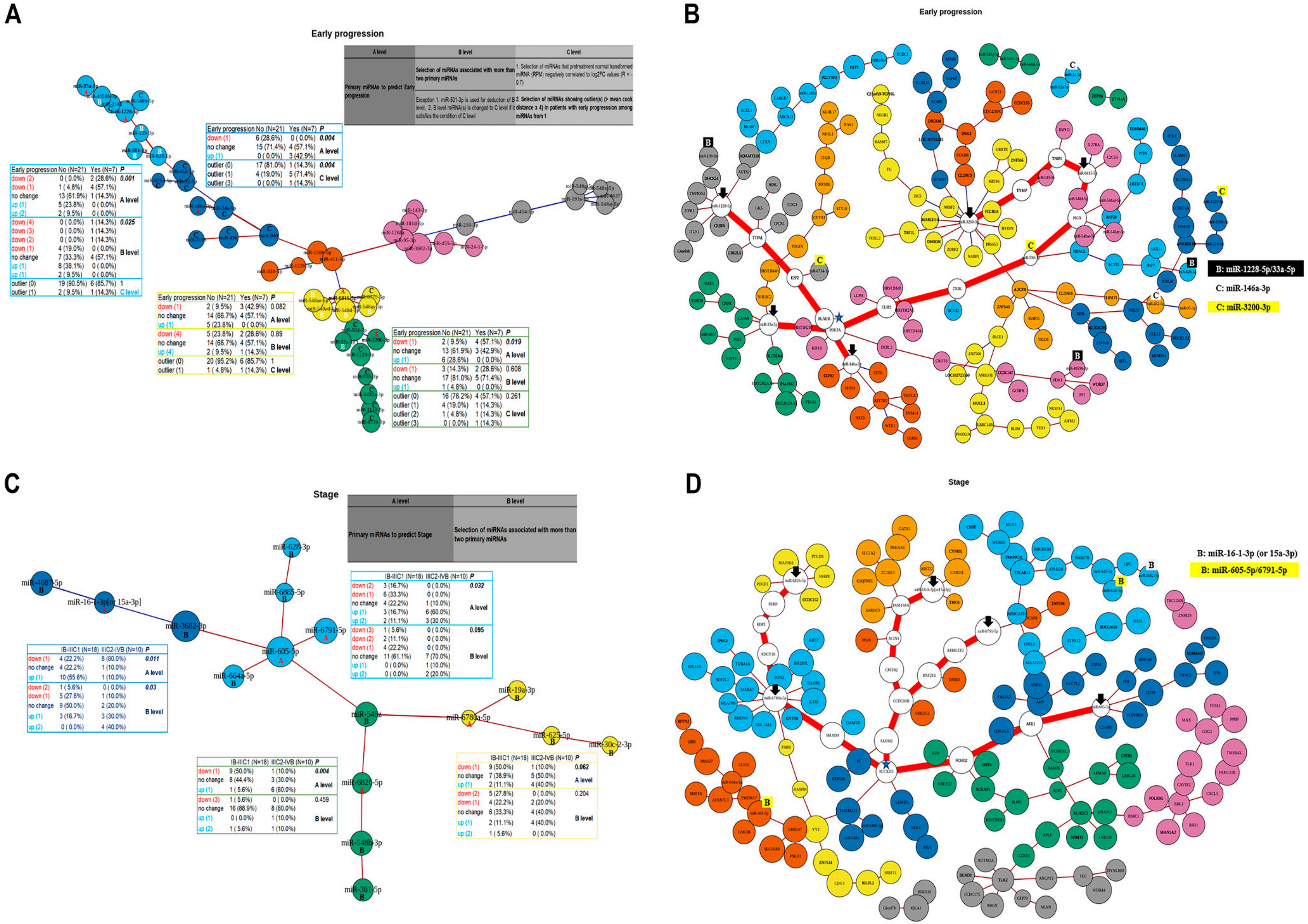
A
Early progression
A level | B level | C level
Primary miRNAs to predict Early progression
Selection of miRNAs associated with more than two primary miRNAs
Exception 1. miR-501-3p is used for deduction of B level, 2. B level miRNA(s) is changed to C level if it satisfies the condition of C level
1. Selection of miRNAs that pretreatment normal transformed miRNA (RPM) negatively correlated to log2FC values (R < -0.7)
2. Selection of miRNAs showing outlier(s) (> mean cook distance x 4) in patients with early progresssion among miRNAs from 1
Early progression No (N=21) Yes (N=7) P
down (1) 6 (28.6%) 0 (0.0%) 0.004
no change 15 (71.4%) 4 (57.1%) A level
up (1) 0 (0.0%) 3 (42.9%)
outlier (0) 17 (81.0%) 1 (14.3%) 0.004
outlier (1) 4 (19.0%) 5 (71.4%) C level
outlier (3) 0 (0.0%) 1 (14.3%)
Early progression No (N=21) Yes (N=7) P
down (2) 0 (0.0%) 2 (28.6%) 0.001
down (1) 1 (4.8%) 4 (57.1%)
no change 13 (61.9%) 1 (14.3%) A level
up (1) 5 (23.8%) 0 (0.0%)
up (2) 2 (9.5%) 0 (0.0%)
down (4) 0 (0.0%) 1 (14.3%) 0.025
down (3) 0 (0.0%) 1 (14.3%)
down (2) 0 (0.0%) 1 (14.3%)
down (1) 4 (19.0%) 0 (0.0%) B level
no change 7 (33.3%) 4 (57.1%)
up (1) 8 (38.1%) 0 (0.0%)
up (1) 2 (9.5%) 0 (0.0%)
outlier (0) 19 (90.5%) 6 (85.7%) 1
outlier (1) 2 (9.5%) 1 (14.3%) C level
Early progression No (N=21) Yes (N=7) P
down (1) 2 (9.5%) 3 (42.9%) 0.082
no change 14 (66.7%) 4 (57.1%) A level
up (1) 5 (23.8%) 0 (0.0%)
down (4) 5 (23.8%) 2 (28.6%) 0.89
no change 14 (66.7%) 4 (57.1%) B level
up (4) 2 (9.5%) 1 (14.3%)
outlier (0) 20 (95.2%) 6 (85.7%) 1
outlier (1) 1 (4.8%) 1 (14.3%) C level
Early progression No (N=21) Yes (N=7) P
down (1) 2 (9.5%) 4 (57.1%) 0.019
no change 13 (61.9%) 3 (42.9%) A level
up (1) 6 (28.6%) 0 (0.0%)
down (1) 3 (14.3%) 2 (28.6%) 0.608
no change 17 (81.0%) 5 (71.4%) B level
up (1) 1 (4.8%) 0 (0.0%)
outlier (0) 16 (76.2%) 4 (57.1%) 0.261
outlier (1) 4 (19.0%) 1 (14.3%)
outlier (2) 1 (4.8%) 1 (14.3%) C level
outlier (3) 0 (0.0%) 1 (14.3%)
B
Early progression
B: miR-1228-5p/33a-5p
C: miR-146a-3p
C: miR-3200-3p
C
Stage
A level | B level
Primary miRNAs to predict Stage
Selection of miRNAs associated with more than two primary miRNAs
IB-IIIC1 (N=18) IIIC2-IVB (N=10) P
down (2) 3 (16.7%) 0 (0.0%) 0.032
down (1) 6 (33.3%) 0 (0.0%)
no change 4 (22.2%) 1 (10.0%) A level
up (1) 3 (16.7%) 6 (60.0%)
up (2) 2 (11.1%) 3 (30.0%)
down (3) 1 (5.6%) 0 (0.0%) 0.095
down (2) 2 (11.1%) 0 (0.0%)
down (1) 4 (22.2%) 0 (0.0%)
no change 11 (61.1%) 7 (70.0%) B level
up (1) 0 (0.0%) 1 (10.0%)
up (2) 0 (0.0%) 2 (20.0%)
IB-IIIC1 (N=18) IIIC2-IVB (N=10) P
down (1) 4 (22.2%) 8 (80.0%) 0.011
no change 4 (22.2%) 1 (10.0%) A level
up (1) 10 (55.6%) 1 (10.0%)
down (2) 1 (5.6%) 0 (0.0%) 0.03
down (1) 5 (27.8%) 1 (10.0%)
no change 9 (50.0%) 2 (20.0%) B level
up (1) 3 (16.7%) 3 (30.0%)
up (2) 0 (0.0%) 4 (40.0%)
IB-IIIC1 (N=18) IIIC2-IVB (N=10) P
down (1) 9 (50.0%) 1 (10.0%) 0.004
no change 8 (44.4%) 3 (30.0%) A level
up (1) 1 (5.6%) 6 (60.0%)
down (3) 1 (5.6%) 0 (0.0%) 0.459
no change 16 (88.9%) 8 (80.0%) B level
up (1) 0 (0.0%) 1 (10.0%)
up (2) 1 (5.6%) 1 (10.0%)
IB-IIIC1 (N=18) IIIC2-IVB (N=10) P
down (1) 9 (50.0%) 1 (10.0%) 0.062
no change 7 (38.9%) 5 (50.0%) A level
up (1) 2 (11.1%) 4 (40.0%)
down (2) 5 (27.8%) 0 (0.0%) 0.204
down (1) 4 (22.2%) 2 (20.0%)
no change 6 (33.3%) 4 (40.0%) B level
up (1) 2 (11.1%) 4 (40.0%)
up (2) 1 (5.6%) 0 (0.0%)
miR-4687-5p
miR-16-1-3p[or 15a-3p]
miR-3682-3p
miR-628-3p
miR-6885-5p
miR-6791-5p
miR-605-5p
miR-664a-5p
miR-548z
miR-6780a-5p
miR-19a-3p
miR-625-5p
miR-30c-2-3p
miR-6826-5p
miR-548h-3p
miR-361-5p
D
Stage
B: miR-16-1-3p (or 15a-3p)
B: miR-605-5p/6791-5p

Figure S1. Identification of the network structure formed by selected miRNAs and adjacent RNAs (related to Figure 3)

To understand the network structure surrounding the primary miRNAs (A level) associated with EP, we first selected miRNAs directly associated with them (B level; |R| > 0.6). In addition, we selected miRNAs with outlier(s), with linear regression (> mean Cook's distance × 4) in patients with EP, among miRNAs with log_2FC negatively correlated (R < -0.7) with RPM (C level). We adopted this approach to include the miRNAs associated with homeostasis following irradiation. Therefore, networks were formed consisting of three groups of miRNAs (|R| between edges ≥ 0.4), and whether the expression of A, B, or C level miRNAs were significantly altered according to EP was observed (Fig. 3A). Interestingly, three communities related to miR-1228-5p/33a-5p, miR-3200-3p, and miR-6815-5p showed higher downregulation with respect to the A level in EP group, whereas one community associated with miR-146a-3p showed the opposite, i.e., upregulation of A level miRNAs in the EP compared to that in the non-EP group. Thus, each A-level miRNA independently contributed to EP. Additionally, the association with miR-1228-5p/33a-5p showed higher downregulation with respect to the B level in EP group, whereas that with miR-146a-3p showed more outliers in the C level in EP group. Therefore, two communities, including miR-1228-5p, miR-33a-5p, and miR-146a-3p, which are close to each other in the network, may particularly influence many miRNAs relevant to EP, which however, is not true for the two communities including miR-3200-3p and miR-6815-5p. In order to understand miRNA-mRNA interactions, we further performed network analysis, including mRNAs relevant to the primary miRNAs (|R| > 0.6) and all miRNAs included in Fig. 3A (Fig. 3B; |R| between edges ≥ 0.6). Importantly, when the shortest distance to connect A level miRNAs was calculated, *PDE3A*, centralizing miR-1228-5p, miR-146a-3p, and miR-33a-5p was connected to miR-6815-5p and miR-3200-3p. The 10 RNAs behind the links were *TTPAL*, *E2F2*, *PLAUR*, *PDE3A*, *CLIP2*, *TNIK*, miR-590-3p,

PIGX, *TNIP1*, and *TYMP*. The observation of several B or C level miRNAs in this network validated the structure of miRNA-mRNA as appropriate.

Regarding the stage, we only focused on the A and B levels in extrapelvic metastasis (Fig. 3C). Three communities associated with miR-605-5p/6791-5p, miR-6826-5p, and miR-6780a-5p included more upregulated A level miRNAs in the extrapelvic metastasis group, whereas the community with miR-16-1-3p [or 15a-3p] included more downregulated A level miRNAs in the extrapelvic metastasis group. Thus, each A level miRNA independently contributed to metastasis. Additionally, the two communities related to miR-605-5p/6791-5p and miR-16-1-3p[or 15a-3p] showed more upregulated B level miRNAs in the extrapelvic metastasis group; therefore, these two communities, close to each other, may be a large group influencing the expression of many miRNAs relevant to metastasis. Next, we assessed miRNA-mRNA interactions (Fig. 3D), and found *PLCXD3* to be the mRNA centralizing primary miRNAs. Fifteen mRNAs, namely *F5, FAM168A, ACIN1, CMTR2, CCDC88B, RNF216, ARHGEF2, ALDH2, PLCXD3, FCHO2, ATE1, SMAD9, ADCY10, RBP3,* and *PERP* were defined as relevant.

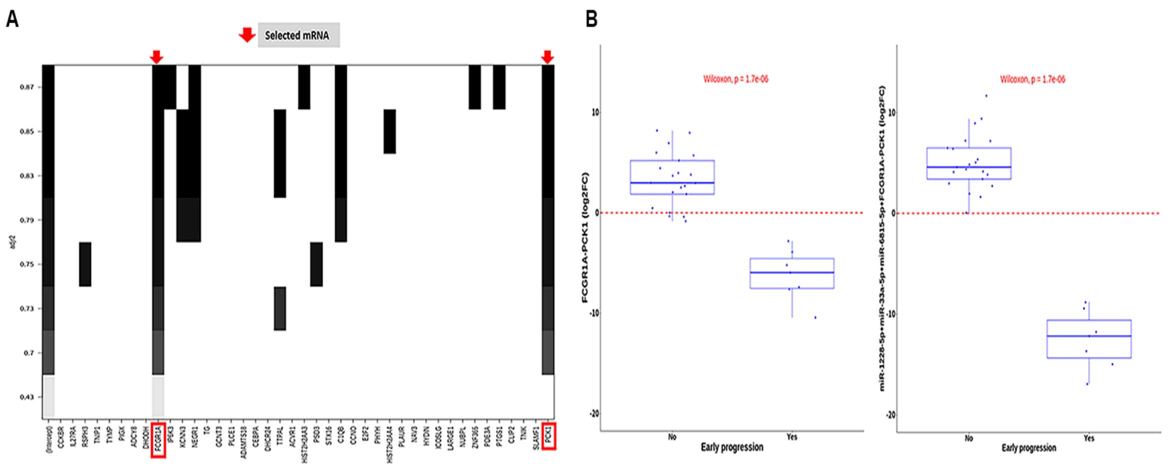

Figure S2. Selection of mRNAs associated with early progression in a simplified network (related to Figure 4)

(A) *PCK1* and *FCGR1A*, with high adjusted R values, were revealed through possible combinations of 41 mRNAs from the network in Figure 4A.

(B) Differences in *FCGR1A-PCK1* (log_2FC) according to early progression were less than that of miR-1228-5p+miR-33a-5p+miR-6815-5p+FCGR1A-PCK1 (log_2FC). P-values were determined using the Wilcoxon rank-sum test.

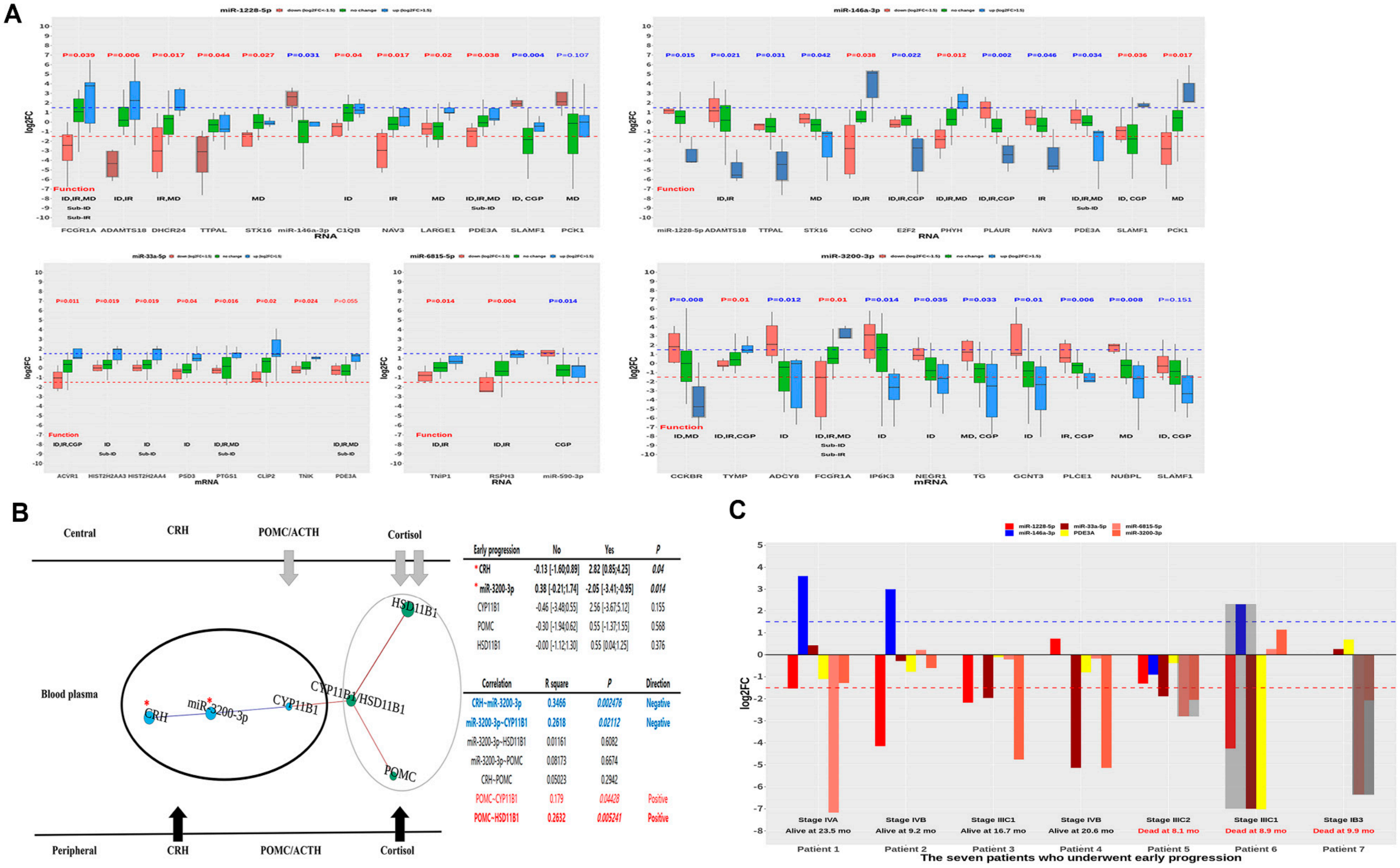

Early progression	No	Yes	*P*
* CRH	-0.13 [-1.60;0.89]	2.82 [0.85;4.25]	*0.04*
* miR-3200-3p	0.38 [-0.21;1.74]	-2.05 [-3.41;-0.95]	*0.014*
CYP11B1	-0.46 [-3.48;0.55]	2.56 [-3.67;5.12]	0.155
POMC	-0.30 [-1.94;0.62]	0.55 [-1.37;1.55]	0.568
HSD11B1	-0.00 [-1.12;1.30]	0.55 [0.04;1.25]	0.376

Correlation	R square	*P*	Direction
CRH~miR-3200-3p	0.3466	*0.002476*	Negative
miR-3200-3p~CYP11B1	0.2618	*0.02112*	Negative
miR-3200-3p~HSD11B1	0.01161	0.6082	
miR-3200-3p~POMC	0.08173	0.6674	
CRH~POMC	0.05023	0.2942	
POMC~CYP11B1	0.179	*0.04428*	Positive
POMC~HSD11B1	0.2632	*0.005241*	Positive

Figure S3. Details of simplified network of miRNA-mRNA interactions in early progression (related to Figure 4)

(A) Boxplots representing the RNAs altered by the five miRNAs; the interquartile range of $\log_2$FC > 1.5 is indicated by the shadow. The p values are marked above boxplots in red (positive correlation) or blue (negative correlation), and in bold ($P < 0.05$) or plain ($P \geq 0.05$) fonts. Functional categories are defined below the boxplots as ID (inflammatory disease), IR (inflammatory response), MD (metabolic disease), CGP (cellular growth and proliferation), Sub-ID (severe inflammatory disorder), and Sub-IR (antigen presentation in macrophages).

(B) Network and linear correlations across *CRH, POMC, CYP11B1, HSD11B1*, and miR-3200-3p and the differences in $\log_2$FC between the two groups according to early progression.

(C) Bar graphs representing miR-1228-5p, miR-146a-3p, miR-33a-5p, PDE3A, miR-6815-5p, and miR-3200-3p from 7 patients showing early progression; the stage, survival status, and follow-up duration are indicated.

Statistical analysis was performed using the Wilcoxon rank-sum test or Kruskal-Wallis test. Downregulation and upregulation of RNAs refer to $\log_2$FC < -1.5 and $\log_2$FC > 1.5, respectively.

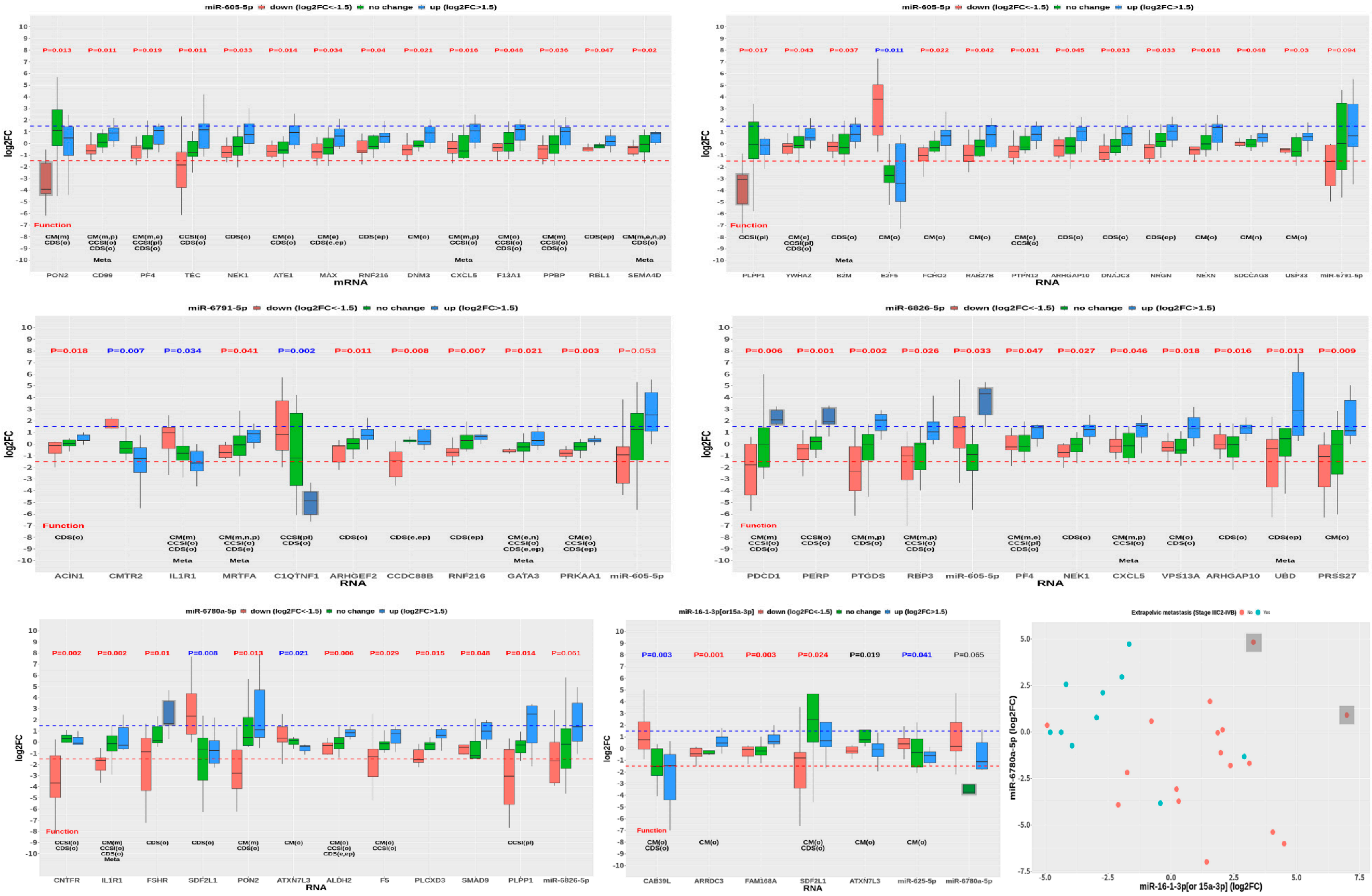
miR-605-5p down (log2FC<-1.5) no change up (log2FC>1.5)
P=0.013 P=0.011 P=0.019 P=0.011 P=0.033 P=0.014 P=0.034 P=0.04 P=0.021 P=0.016 P=0.048 P=0.036 P=0.047 P=0.02
Function
CM(m) CDS(o)
CM(m,p) CCSI(o) CDS(o)
CM(m,e) CCSI(pl) CDS(o)
CCSI(o) CDS(o)
CDS(o)
CM(o) CDS(o)
CM(e) CDS(e,ep)
CDS(ep)
CM(o)
CM(m,p) CCSI(o)
CM(o) CCSI(o) CDS(o)
CM(m) CCSI(o) CDS(o)
CDS(ep)
CM(m,e,n,p) CDS(o)
Meta
PON2 CD99 PF4 TEC NEK1 ATE1 MAX RNF216 DNM3 CXCL5 F13A1 PPBP RBL1 SEMA4D
mRNA
log2FC
miR-605-5p down (log2FC<-1.5) no change up (log2FC>1.5)
P=0.017 P=0.043 P=0.037 P=0.011 P=0.022 P=0.042 P=0.031 P=0.045 P=0.033 P=0.033 P=0.018 P=0.048 P=0.03 P=0.094
Function
CCSI(pl)
CM(e) CCSI(pl) CDS(o)
CDS(o)
CM(o)
CM(o)
CM(o)
CM(e) CCSI(o)
CDS(o)
CDS(o)
CDS(ep)
CM(o)
CM(n)
CM(o)
Meta
PLPP1 YWHAZ B2M E2F5 FCHO2 RAB27B PTPN12 ARHGAP10 DNAJC3 NRGN NEXN SDCCAG8 USP33 miR-6791-5p
RNA
miR-6791-5p down (log2FC<-1.5) no change up (log2FC>1.5)
P=0.018 P=0.007 P=0.034 P=0.041 P=0.002 P=0.011 P=0.008 P=0.007 P=0.021 P=0.003 P=0.053
Function
CDS(o)
CM(m) CCSI(o) CDS(o)
CM(m,n,p) CCSI(o) CDS(e)
CCSI(pl) CDS(o)
CDS(o)
CDS(e,ep)
CDS(ep)
CM(e,n) CCSI(o) CDS(e,ep)
CM(e) CCSI(o) CDS(ep)
Meta
ACIN1 CMTR2 IL1R1 MRTFA C1QTNF1 ARHGEF2 CCDC88B RNF216 GATA3 PRKAA1 miR-605-5p
RNA
miR-6826-5p down (log2FC<-1.5) no change up (log2FC>1.5)
P=0.006 P=0.001 P=0.002 P=0.026 P=0.033 P=0.047 P=0.027 P=0.046 P=0.018 P=0.016 P=0.013 P=0.009
Function
CM(m) CCSI(o) CDS(o)
CCSI(o) CDS(o)
CM(m,p) CDS(o)
CM(m,p) CCSI(o) CDS(o)
CM(m,e) CCSI(pl) CDS(o)
CDS(o)
CM(m,p) CCSI(o)
CM(o) CDS(o)
CDS(o)
CDS(ep)
CM(o)
Meta
PDCD1 PERP PTGDS RBP3 miR-605-5p PF4 NEK1 CXCL5 VPS13A ARHGAP10 UBD PRSS27
RNA
miR-6780a-5p down (log2FC<-1.5) no change up (log2FC>1.5)
P=0.002 P=0.002 P=0.01 P=0.008 P=0.013 P=0.021 P=0.006 P=0.029 P=0.015 P=0.048 P=0.014 P=0.061
Function
CCSI(o) CDS(o)
CM(m) CCSI(o) CDS(o)
CDS(o)
CDS(o)
CM(m) CDS(o)
CM(o)
CM(o) CCSI(o) CDS(e,ep)
CM(o) CCSI(o)
CCSI(pl)
Meta
CNTFR IL1R1 FSHR SDF2L1 PON2 ATXN7L3 ALDH2 F5 PLCXD3 SMAD9 PLPP1 miR-6826-5p
RNA
miR-16-1-3p[or15a-3p] down (log2FC<-1.5) no change up (log2FC>1.5)
P=0.003 P=0.001 P=0.003 P=0.024 P=0.019 P=0.041 P=0.065
Function
CM(o) CDS(o)
CM(o)
CM(o) CDS(o)
CM(o)
CM(o)
CAB39L ARRDC3 FAM168A SDF2L1 ATXN7L3 miR-625-5p miR-6780a-5p
RNA
Extrapelvic metastasis (Stage IIIC2-IVB) No Yes
miR-6780a-5p (log2FC)
miR-16-1-3p[or 15a-3p] (log2FC)
5.0 2.5 0.0 -2.5 -5.0 -7.5
-5.0 -2.5 0.0 2.5 5.0 7.5

Figure S4. Details of simplified network of miRNA-mRNA interactions in metastasis (related to Figure 5)

Boxplots representing the RNAs altered by the five miRNAs; the interquartile range of $log_2FC > 1.5$ is indicated by the shadow. miR-16-1-3p [or 15a-3p] and miR-6780a-5p are marked as two outliers. The p values are marked above boxplots in red (positive correlation) or blue (negative correlation), and in bold ($P < 0.05$) or plain ($P \geq 0.05$) fonts. The functional categories are defined below the boxplots as CM (cellular movement), CCSI (cell to cell signaling and interaction), CDS (cell death and survival), Meta (metastasis of tumor cell lines), m (myeloid cells), e (endothelial cells), n (neurons), p (phagocytes), o (others), pl (platelets), and ep (epithelial cells).

Statistical analysis was performed using the Wilcoxon rank-sum test or Kruskal-Wallis test. Downregulation and upregulation of RNAs refer to $log_2FC < -1.5$ and $log_2FC > 1.5$, respectively.

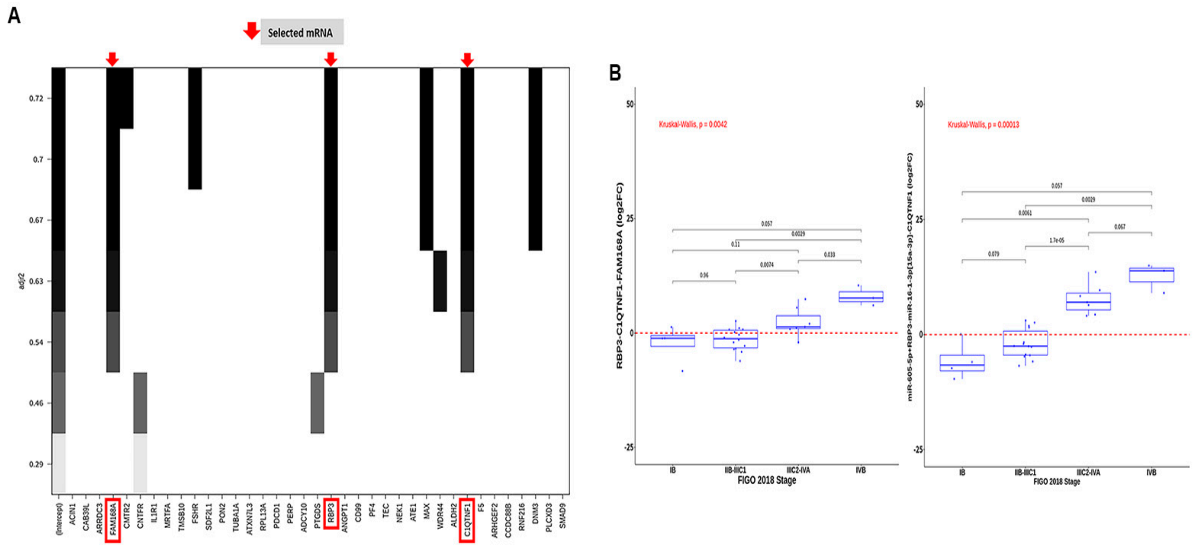

Figure S5. **Selection of mRNAs associated with metastasis in the simplified network (related Figure 5)**

(A) *FAM168A*, *RBP3*, and *C1QTNF1*, with high adjusted R values, were revealed through possible combinations of 37 mRNAs from five communities, including the main miRNAs from the network in Figure 5A.

(B) Differences in RBP3-C1QTNF1-FAM168A (log_2FC) according to stage were lower than that in miR-605-5p+RBP3-miR-16-1-3p [or 15a-3p]-C1QTNF1 (log_2FC). *P*-values were determined using the Wilcoxon rank-sum test.

Table S1. **Candidate RNAs for the ingenuity pathway analysis (related to Figure 3)**

A. mRNAs closely related to each clinical factor

Clinical factor	Correlation coefficient	Gene symbol (10)
Early progression	|R| > 0.6	PCK1, PRR29, PRMT8, STX16, DERL2, PFKFB3, THOC6, PRMT6, FCGR1A, PPP1R35
Clinical factor	Correlation coefficient	Gene symbol (16)
Stage & Stage IIIC2-IVB	|R| > 0.5 & |R| > 0.5	PTGDS, TRIM50, TCTA, DIO1, GALNTL5, GSTM1, TRIM29, MEOX2, RBP3, WDR60, PSMD9, TIAF1, PRICKLE1, C3orf14, HTRA4, BLID

B. miRNAs and mRNAs obtained through network analysis using miRNAs and mRNAs adjacent to primary miRNAs (Figure S1B and S1D)

Ingenuity pathway analysis using miRNAs and mRNAs from A and B
